# Species Identification and Clarithromycin Susceptibility Testing of 278 Clinical Nontuberculosis Mycobacteria Isolates

**DOI:** 10.1155/2015/506598

**Published:** 2015-06-01

**Authors:** Wenjuan Nie, Hongfei Duan, Hairong Huang, Yu Lu, Naihui Chu

**Affiliations:** ^1^Department of Tuberculosis, Beijing Chest Hospital, Capital Medical University, Tongzhou District, Beijing 101149, China; ^2^Reference Lab, Beijing Chest Hospital, Capital Medical University, Tongzhou District, Beijing 101149, China; ^3^Department of Pharmacology, Beijing Chest Hospital, Capital Medical University, Tongzhou District, Beijing 101149, China

## Abstract

Purpose of this paper is to analyze different species' proportion of nontuberculosis mycobacteria (NTM) and susceptibility to clarithromycin of different species. 278 clinical NTM isolates were identified into species by using 16S rRNA, *rpoB* and *hsp65*. Then clarithromycin susceptibility testing against different species was done separately, using microplate Alamar Blue assay. Finally, resistance isolates' *erm(41)* of *M. abscessus* were sequenced in order to analyze mechanisms for clarithromycin resistant. In this test, 131 isolates (47%) belonged to *M. avium* complex (MAC), and 70 isolates (25%) belonged to *M. abscessus*. Nearly all the *M. abscessus* subsp. *abscessus* resistant to clarithromycin had T28 in *erm(41)*. However, all the *M. abscessus* subsp. *abscessus* susceptible to clarithromycin had C28 in *erm(41)*. In this study, we find that MAC was the most common pathogens of NTM, and the second one was *M. abscessus*. However, *M. chelonei, M. fuerth*, and *M. gordon* were rare. Clarithromycin had a good inhibition activity against all the NTM species except *M. abscessus* subsp. *abscessus*. The *erm(41)* genotype is of high relevance to clarithromycin resistance.

## 1. Introduction

Nontuberculosis mycobacteria (NTM) are globally recognized as pathogens especially in immunocompromised population including HIV/AIDS (Human Immunodeficiency Virus/Acquired Immunodeficiency Syndrome) patients. In addition to causing infections in immune-compromised patients, this group of organisms has been reported to be increasingly recognized as pathogens from immunocompetent individuals as well [1]. Different species have different susceptibility to antibiotics, so it is important to identify NTM into different species in order to make the respective therapy plan. The American Thoracic Society (ATS) and the Infectious Diseases Society of America (IDSA) recommended treatment for NTM pulmonary and disseminated disease is a macrolide-containing, multidrug regimen. And clarithromycin, a macrolide drug, is described as the key therapy for NTM diseases [2]. Clarithromycin forms the cornerstone of NTM treatment and is the only drug recommended by the Clinical and Laboratory Standards Institute (CLSI) for drug susceptibility testing (DST) [3]. However, antibiotic treatment of pulmonary* M. abscessus* infections remains problematic. Infections due to the* M. abscessus* group are difficult to treat because these mycobacteria are intrinsically resistant not only to the classical antituberculous drugs but also to most of the antibiotics that are currently available. Clarithromycin had been the drug of choice for* M. abscessus* infections for the past 20 years before a resistance gene,* erm(41)*, was described in* M. abscessus*. The discovery of a novel inducible* erm* gene,* erm(41)*, provides an explanation for the lack of efficacy for macrolides against* M. abscessus* infections [4]. A polymorphism in* erm(41)* (T or C) was described at position 28 and its association with the susceptibility was examined. Our objective was to identify NTM isolates into different species and to do the DST separately and then to seek the presence of* erm(41)* in a large collection of clinical strains precisely identified as* M. abscessus* and finally to correlate these results with clarithromycin susceptibility.

## 2. Materials and Methods

### 2.1. Species Identification

All of the 278 clinical strains in Reference Lab of Beijing Chest Hospital were isolated from sputum samples of patients, which were previously identified as NTM strain using growth experiments of p-nitrobenzoic acid (PNB) by Reference Lab of Beijing Chest Hospital. All the strains were subcultured on the Lowenstein-Jensen medium at 37°C to observe colony morphology. The incubation time ranged from 4 to 28 days. Then colonies were used for species identification based on 16S rRNA sequence analysis. Preparation of the PCR reaction mixture and amplification were done as described previously [5, 6]. PCR products were purified and sequenced by the BGI corporation, using forward and reverse primers. Both strands were sequenced as a crosscheck. Species identification of these strains was accomplished by the sequencing of 16S rRNA, using BLAST search to measure the similarities.

### 2.2. Drug Susceptibility Testing

We performed DST of NTM isolates by broth microdilution using 96-well plates, which is a reference method as recommended by CLSI [3]. DST of these NTM isolates was done as described previously [6]. To* M. abscessus*, plates were submitted to an extended incubation of 14 days' incubation at 30°C. However, to other NTM isolates, the incubation time ranged from 3 to 10 days. A color change from blue to pink indicates bacterial growth. MIC was defined as the lowest concentration of the drug that showed no color change, which was the lowest concentration of drug capable of inhibiting the visible growth of tested isolates. MIC_50_ and MIC_90_ values were defined as drug concentrations, which inhibit 50% and 90% of isolates, respectively. Susceptibility was evaluated according to CLSI breakpoint recommendations [3]. The type strains of American Type Culture Collection (ATCC) were used for comparison.

### 2.3. Clarithromycin Resistant Mechanism

Crude DNA preparations suitable for PCR were obtained by heating suspensions of mycobacteria in Tris-EDTA buffer at 100°C for 15 to 20 min. Debris was removed from the preparations by centrifugation at 16,000 rpm for 3 min. The basic 40 *μ*L PCR mixture comprised 2 *μ*L DNA, 2 *μ*L DNA polymerase, and 1 *μ*L each primer and finally was complete to 40 *μ*L by double distilled water. Reactions were run for 3 min at 94°C, followed by 30 cycles at 94°C for 1 min, 61°C for 1 min, and 72°C for 1 min. For cloning of* erm(41)*, gene segments were amplified with primers 5-ACG TTG GAT CCG AGC GCC GTC ACA AGA TGC ACA-3 and 5-GCG AGA AGC TTG ACT TCC CCG CAC CGA TTC CAC-3. The resulting amplification products were purified and sequenced by the BGI corporation, using forward and reverse primers. Both strands were sequenced as a crosscheck. The sequence results were compared with* M. abscessus* standard strains.

## 3. Result

### 3.1. Species Identification

Of all the 278 NTM clinical strains (Table 1), 100 (36%) isolates belonged to rapidly growing* Mycobacterium* (RGM), and 178 (64%) isolates belonged to slowly growing* Mycobacterium* (SGM). Among these species, 70 isolates (25%) belonged to* M. abscessus*, 23 isolates (8%) belonged to* M. fortuitum*, 5 isolates (2%) belonged to* M. chelonei*, 2 isolates (1%) belonged to* M. fuerth*, 131 isolates (47%) belonged to* M. avium* complex (MAC), 34 isolates (12%) belonged to* M. kansasii*, and 13 isolates (5%) belonged to* M. gordon*. Of the 70* M. abscessus* strains, according to* rpoB* and* hsp65*, 45 strains (64%) belonged to* M. abscessus* subsp.* abscessus*, and 25 strains (36%) belonged to* M. abscessus* subsp.* massiliense*.

All the 70* M. abscessus* isolates were divided into two subspecies,* M. abscessus* subsp.* abscessus* and* M. abscessus* subsp.* massiliense* (Table 2). There was no* M. abscessus* subsp.* bolletii* strain observed. There were two ways to identify subspecies: sequencing* rpoB* PCR-restriction fragments and sequencing* hsp65* PCR-restriction fragments. Results of these two methods were the same (*M. abscessus* subsp.* abscessus*, 64%;* M. abscessus* subsp.* massiliense*, 36%), which means that all the* M. abscessus* subsp.* abscessus* and* M. abscessus* subsp.* massiliense* strains divided by* rpoB* sequence were all verified by* hsp65* sequence. There was no discordant result between* rpoB* and* hsp65* sequences analysis. The* rpoB* similarity of* M. abscessus* subsp.* abscessus* to the reference sequences was 98% to 100%, and the* hsp65* similarity was 98% to 100%. The* rpoB* similarity of* M. abscessus* subsp.* massiliense* to the reference sequences was relatively low, 97% to 100%, and the* hsp65* similarity was 98% to 100%.

### 3.2. Drug Susceptibility Testing

The breakpoint, MIC range, MIC_50_, MIC_90_, and proportions of susceptible, moderately susceptible, and resistant strains of each drug against NTM are shown in Table 3, respectively. As RGM,* M. fortuitum* should be incubated for 3 days (CLSI recommends), and then the MIC value is read. In Table 3, it shows that most of* M. fortuitum* (92%) strains were susceptible to clarithromycin, and the resistant proportion was low (4%). Although* M. abscessus* belong to RGM, the new CLSI guideline extended the incubation time to 14 days, which is the shortest time CLSI recommends for final reading. In this test,* M. abscessus* was divided into different subspecies,* M. abscessus* subsp.* abscessus* and* M. abscessus* subsp.* massiliense*, and the DST was done separately. It appeared that most of the* M. abscessus* subsp.* massiliense* strains are susceptible to clarithromycin, whereas susceptibility was less clear for* M. abscessus* subsp.* abscessus*, with most strains being resistant and others susceptible. In Table 3, it shows that most* M. abscessus* subsp.* massiliense* (96%) strains were susceptible to clarithromycin, and the resistant proportion was low (4%). However, the other subspecies,* M. abscessus* subsp.* abscessus*, had an entirely different result. Most of* M. abscessus* subsp.* abscessus* (83%) strains were resistant to clarithromycin, and there were only 10% susceptible to it. As SGM, MAC had the biggest proportion of isolates, and the number was 131 isolates. Similarly, most MAC (95%) were susceptible to clarithromycin, and the resistant proportion was low (4%).* M. Kansasii* had the highest susceptible proportion. All of them (100%) were susceptible to clarithromycin. The sample size of* M. chelonei* and* M. fuerth* was small, so it is inappropriate to calculate the MIC_50_, MIC_90_, and proportions of susceptible, moderately susceptible, and resistant strains.  Table 4 displays each isolate's MIC value of* M. chelonei* and* M. fuerth*. All of them were susceptible to clarithromycin.

### 3.3. Clarithromycin Resistant Mechanism

*erm(41)* genes in subspecies of* M. abscessus* showed characteristic differences. Compared with its homologues in* M. abscessus* subsp.* abscessus*, the* erm(41)* in* M. abscessus* subsp.* massiliense* maybe dysfunctional due to deletion of nucleotides. For 49 isolates, the* erm(41)* gene segment was sequenced successfully. Nine isolates belonged to* M. abscessus* subsp.* massiliense*, and all the* erm(41)* of which were incomplete. The rest 40 isolates belonged to* M. abscessus* subsp.* abscessus*, and all the* erm(41)* of which were complete. After analyzing these* erm(41)* gene segments, it showed that (Table 5) except 1* M. abscessus* subsp.* abscessus* isolate which had C28 in* erm(41)*, nearly all the* M. abscessus* subsp.* abscessus* strains resistant to clarithromycin had T28 in* erm(41)*. However, all the* M. abscessus* subsp.* abscessus* strains susceptible to clarithromycin had C28 in* erm(41)*. It shows that* erm(41)* sequence differentiated these two subspecies, and its specific features were predictive for clarithromycin susceptibility or resistance.

## 4. Discussion

### 4.1. Species Identification

The proportions of different species are variable, according to geographical distribution. Geographical variation occurs in the pattern of NTM isolation across different countries.* M. fortuitum* was the commonest species reported from Iran and Turkey whereas MAC predominated most of the European countries and Brazil. In Korea,* M. abscessus* is the second most common pathogen responsible for lung disease caused by NTM, following the MAC [7]. This is the first study to focus primarily on the clinical relevance of the species differentiation among NTM in Beijing, and the largest study of its kind, with almost 278 patients' sputum samples included. In this test, SGM had the larger proportion of NTM than RGM. Among SGM, MAC had the largest proportion in NTM. Nearly half of NTM belonged to MAC. And the second-largest proportion of NTM was* M. abscessus*. Nearly a quarter of NTM belonged to* M. abscessus*. This result is similar to Korea's [8].* M. chelonei*,* M. fuerth,* and* M. gordon*'s proportion was small. There were lots of special species, including* M. chelonei*,* M. fuerth,* and* M. gordon*, which had only few amounts, so it is difficult to calculate and analyze the characteristics of these rare species* in vitro*. In the future we should collect more samples to assess these special species in order to guide clinical treatment.

Two new* M. abscessus*-related species,* M. abscessus* subsp.* massiliense* and* M. abscessus* subsp.* bolletii*, were identified, which were previously grouped with* M. abscessus* [5]. Among 40 patients monitored at the National Institutes of Health (Bethesda, MD), the prevalence of* M. abscessus* subsp.* massiliense* and* M. abscessus* subsp.* bolletii* was 28% and 5%, respectively [9]. In the Netherlands, 21% of 39 clinical isolates of* M. abscessus* were identified as* M. abscessus* subsp.* massiliense* and 15% were* M. abscessus* subsp.* bolletii* [10]. In France,* M. abscessus* subsp.* massiliense* and* M. abscessus* subsp.* bolletii* accounted for 22 and 18% of 50 patients with cystic fibrosis infected with* M. abscessus*, respectively [11]. In Korea,* M. abscessus* subsp.* abscessus* and* M. abscessus* subsp.* massiliense* are isolated in almost equal numbers among* M. abscessus* infections, whereas* M. abscessus* subsp.* bolletii* is rare. A previous study showed that nearly half (47%) of all* M. abscessus* clinical isolates were identified as* M. abscessus* subsp.* massiliense*, although the prevalence of* M. abscessus* subsp.* bolletii* was low (2%) [5]. Our test has the same result as Korea's. In this test, more than half* M. abscessus* belonged to* M. abscessus* subsp.* abscessus* and a little less than a half belonged to* M. abscessus* subsp.* massiliense*. There was no one* M. abscessus* subsp.* bolletii* observed in our test, which means that* M. abscessus* subsp.* bolletii* accounts for a very small proportion of* M. abscessus*. Differentiation among* M. abscessus* subsp.* abscessus*,* M. abscessus* subsp.* massiliense*, and* M. abscessus* subsp.* bolletii* is challenging for clinical microbiology laboratories. Recent work proved the inaccuracy of single-target sequencing for separating these three taxa within the* M. abscessus* group. In some strains, additional housekeeping genes were analyzed because of the discordant results between* rpoB* and* hsp65* genes analysis [5, 12]. Sequencing of more other targets (such as* secA1*,* soda,* and ITS), combined with phylogenomic analysis, has been shown to increase identification accuracy among these different taxa [8]. More recently, a multilocus sequence analysis targeting 8 housekeeping genes and a multispacer sequence analysis were reported [13, 14]. However, all of these methods require genomic sequencing, which is relatively costly and time consuming and may not be available in all clinical microbiology laboratories.

### 4.2. Drug Susceptibility Testing

NTM infection is a refractory disease that, unlike tuberculosis, does not respond well to conventional antituberculosis drug. With the progress of mycobacterial taxonomy and speciation, it appeared that antibiotic susceptibility of NTM is tightly related to mycobacterial species and, conversely, that a homogenous intrinsic susceptibility pattern may reflect the homogeneity of the species [2, 7]. Since many new species have been described recently with the assistance of molecular biology and genomics, we need to assess their intrinsic susceptibility patterns species by species. The macrolide clarithromycin is considered a cornerstone in antimicrobial chemotherapy of NTM infections. Clarithromycin is a 14-membered ring macrolide that binds to the large ribosomal subunit in the vicinity of the peptidyl transferase center and inhibits protein synthesis, which results in the arrest of bacterial growth. Clarithromycin is given orally and is highly active against many species of NTM. It is the only drug of demonstrated efficacy that can be administered orally. In all of the studies so far, most variations were reported in the clarithromycin susceptibility results within different NTM species even with strains precisely identified and even with reference strains [5, 15, 16]. For example,* M. abscessus* subsp.* bolletii* was shown to be clarithromycin resistant by Adékambi and Drancourt [15] and susceptible by Leao et al. [16]. NTM species show a characteristically different response to clarithromycin from others, and precise identification of these species would be important for the treatment of infected patients. This is why we would like to propose that clarithromycin susceptibility be assessed on a molecular basis for all new cases of* M. abscessus* group infections and that the outcomes of clarithromycin therapy be observed as follows. In this study, we evaluated clarithromycin's activity against different NTM species, since this is the single drug CLSI recommended in* in vitro* testing. The CLSI recommends the use of the radiometric BACTEC 460, the broth-based macrodilution, or the broth microdilution method for the testing of NTM susceptibility to clarithromycin, and it requires weeks to complete from the time of sample collection because of the growing rate of organism.

As the most common NTM species, the treatment outcome of MAC pulmonary disease was disappointing before the use of newer macrolide antibiotics such as clarithromycin and azithromycin. Since 1990 introduction of newer macrolide antibiotics, treatment of pulmonary MAC disease has improved patient outcome. The ATS and IDSA recommend that a three-drug regimen be utilized for the treatment of MAC infection, including a macrolide (clarithromycin or azithromycin). The macrolides are the only class of drugs for which a correlation has been demonstrated between* in vitro* susceptibility results and* in vivo* clinical response in MAC disease (Griffith, 2007). For this reason, the ATS only recommends routine susceptibility testing for clarithromycin, the macrolide most commonly used for treating MAC disease (Griffith, 2007). In this test clarithromycin showed an excellent inhibition activity against MAC. 95% MAC were susceptible to it. And the resistance proportion was low. Considering the big sample size of 131 isolates, this result again proves clarithromycin's good inhibition activity against MAC. However, ATS guidelines also reported that, in contrast to* Mycobacterium tuberculosis*, DST maybe of little use for the treatment of MAC infection, although clarithromycin is the only drug for which the results of susceptibility testing should be taken into consideration in the treatment of MAC infection [2]. We need to conduct a prospective clinical study in order to compare efficacy* in vivo* and susceptibility data* in vitro*.

As the second-largest NTM species and one of the most notorious causative agents of disease, drug therapy of* M. abscessus* disease is long, costly, resistant to many antibiotics, and often associated with drug-related toxicities, leading to unsatisfactory treatment results. In the 1990s, clarithromycin became the drug of choice for* M. abscessus* infections, and therapeutic successes were reported. Combination therapy of intravenous amikacin with cefoxitin or imipenem and an oral macrolide has been recommended by the ATS/IDSA and many other experts [2]. However, treatment response rates are not satisfactory and optimal therapeutic regimens and treatment durations are not well established. Two new* M. abscessus*-related species,* M. abscessus* subsp.* massiliense* and* M. abscessus* subsp.* bolletii*, were identified, which were previously grouped with* M. abscessus*, so the previous* M. abscessus* now includes* M. abscessus* subsp.* abscessus*,* M. abscessus* subsp.* massiliense,* and* M. abscessus* subsp.* bolletii*. But the data regarding* in vitro* DST results of these new subspecies are limited. It is reported that* M. abscessus* subsp.* abscessus* and* M. abscessus* subsp.* bolletii* have different clinical characteristics and treatment responses* in vivo* and have different DST result* in vitro* [12, 17]. For instance, an experiment in Japan shows that with* M. abscessus* subsp.* abscessus* bronchiectasia may occur more frequently than with* M. abscessus* subsp.* bolletii in vitro*, and the resistant proportion of imipenem against* M. abscessus* subsp.* abscessus* is lower than* M. abscessus* subsp.* bolletii* [17]. An experiment in Korea shows that* M. abscessus* subsp.* abscessus* may have a worse radiographic response on HRCT and microbiologic response than* M. abscessus* subsp.* bolletii in vitro*, and similarly, the resistant proportion of imipenem against* M. abscessus* subsp.* abscessus* is lower than* M. abscessus* subsp.* bolletii* [12]. In our test, it shows that* M. abscessus* subsp.* abscessus* and* M. abscessus* subsp.* massiliense* displayed different susceptibility to clarithromycin. Most of* M. abscessus* subsp.* abscessus* strains were resistant to clarithromycin. However,* M. abscessus* subsp.* massiliense*'s MIC to clarithromycin is small, and nearly all of the isolates were susceptible to it. From a clinical standpoint, our study has some important therapeutic implications. The inducible resistance to clarithromycin of* M. abscessus* subsp.* abscessus* isolates means that it can be much more difficult to treat* M. abscessus* subsp.* abscessus* lung disease. A much longer duration of intravenous antibiotic therapy and more effective oral antibiotics may be needed to improve treatment outcomes in* M. abscessus* subsp.* abscessus* lung disease. The low MIC for clarithromycin and the absence of inducible resistance to clarithromycin suggest that* M. abscessus* subsp.* massiliense* lung disease may be more effectively treated with a clarithromycin-based antibiotic regimen [12].

*M. kansasii* is the second most common cause of NTM disease in the United States. In our test it is the third-largest species, but the sample size is small. In this test,* M. kansasii* showed an excellent susceptibility to clarithromycin. All the* M. kansasii* isolates were susceptible to it. However, according to ATS and IDSA, routine susceptibility testing of* M. kansasii* isolates is recommended for rifampin only, and the recommended treatment of* M. kansasii* pulmonary disease does not include clarithromycin. But the excellent* in vitro* activity of clarithromycin against* M. kansasii* suggests that these agents may be useful in retreatment regimens.* M. fortuitum* infections are rare, and they are usually susceptible to multiple oral antimicrobial agents. In this test,* M. fortuitum* showed a good susceptibility to clarithromycin. All of the* M. fortuitum* isolates were susceptible to clarithromycin, except 1 isolate resistant to it.* In vitro* susceptibility data are limited because of the extreme fastidiousness of the organism [2]. Optimal therapy is not determined, but ATS and IDSA also recommend that multidrug therapies including clarithromycin appear to be more effective than those without clarithromycin.* M. chelonei* and* M. fuerth* are extremely rare species. In our test, isolates of these two species are very few, so Table 4 only shows every isolate's MIC value. In Table 4, it shows that the MIC value is very low and all the* M. chelone* and* M. fuerth* strains were susceptible to clarithromycin. Although the sample size is small, we may still suppose that clarithromycin may have a good inhibition activity against these two species.

### 4.3. Clarithromycin Resistant Mechanism

For* M. abscessus*, one of the most notorious causative agents of disease, the macrolide clarithromycin is considered as cornerstone in antimicrobial chemotherapy of pulmonary* M. abscessus* infection. Paradoxically, however,* M. abscessus* infections of the lung are often intractable to clarithromycin chemotherapy, although high doses of clarithromycin may lead to clinical improvement in some patients [2, 4]. The lack of efficacy of clarithromycin is puzzling, as pretreatment isolates are usually reported as susceptible to clarithromycin when CLSI procedures are used [3, 4]. Intriguingly, Brown et al. reported that the clarithromycin MIC increased for some* M. abscessus* isolates if the incubations for the susceptibility assays were extended, although these isolates would still be deemed clarithromycin susceptible if an incubation period based on the previous CLSI guidelines was used [4, 18]. Similarly, Nash and colleagues showed that although a strain might appear susceptible after 3 days of* in vitro* incubation [3], due to induction of the synthesis of a methyltransferase [4], the strain was clarithromycin resistant if incubation was extended to 14 days or if the strain was preincubated with clarithromycin. So in 2012, CLSI guideline extends* M. abscessus* DST incubation time from 3 days to 14 days. Although* M. abscessus* subsp.* massiliense* remained a high susceptible proportion after this adjustment, the resistant proportion of* M. abscessus* subsp.* abscessus* increased to a high level. An explanation for the instability of clarithromycin MIC is that* M. abscessus* might have inducible resistance to clarithromycin. Inducible resistance to clarithromycin has been suggested as an explanation for the lack of efficacy of clarithromycin-based treatments against* M. abscessus* infection [12]. An erythromycin ribosomal methylase gene,* erm(41)*, has been identified in several isolates of* M. abscessus* and the presence of the gene was associated with inducible resistance to clarithromycin. The induction of resistance was shown to result from increased expression of* erm(41)* as demonstrated by an increase in* erm(41)* RNA levels after exposure to clarithromycin. The functionality of the methylase is dependent on the nucleotide at position 28 of the* erm(41)* gene. Wild type T28 sequevars show inducible clarithromycin resistance, while C28 sequevars do not [4]. In our test, of all the 40* M. abscessus* subsp.* abscessus* isolates which were sequenced successfully, 35 isolates were resistant to clarithromycin, and 34 isolates in them had T28 in* erm(41)*. However, all the 5* M. abscessus* subsp.* abscessus* isolates which were susceptible to clarithromycin had C28 in* erm(41)*. This result also highlights the resistant mechanism above, which means that* erm(41)* may be of relevance to clarithromycin resistance. There is also 1 isolate which has C28 in* erm(41)* and still resistant to clarithromycin. Maybe there are some other resistant mechanisms needed to be analyzed. Although the therapeutic implications of* erm(41)* require further study, it is anticipated that expression of this gene does confer clinically significant resistance to clarithromycin. And still, the clinical impact of inducible resistance remains to be determined.

## 5. Conclusions

In this study, we find that MAC was the most common pathogen of NTM, and the second one was* M. abscessus*. However,* M. chelonei*,* M. fuerth,* and* M. gordon* were rare. Clarithromycin had a good inhibition activity against all the NTM species except* M. abscessus* subsp.* abscessus. erm(41)* genotype which is likely to be a major determinant of clarithromycin resistance of* M. abscessus*.

## Figures and Tables

**Table 1:** The result of NTM species identification.

	Species	Number (%) of strains
RGM *n* = 100 (36%)	*M. abscessus *	70 (25%)
	*M. fortuitum *	23 (8%)
	*M. chelonei *	5 (2%)
	*M. fuerth *	2 (1%)

SGM *n* = 178 (64%)	MAC	131 (47%)
	*M. kansasii *	34 (12%)
	*M. gordon *	13 (5%)

Total *n* = 278 (100%)	NTM	278 (100%)

**Table 2:** Each subspecies' proportion identified by using *rpoB* and *hsp65*.

Sequence analysis	Number (%) of *M. abscessus *subsp.* abscessus *	Number (%) of *M. abscessus *subsp.* massiliense *	Total number (%)
*rpoB *	45 (64%)	25 (36%)	70 (100%)
*hsp65 *	45 (64%)	25 (36%)	70 (100%)

**Table 3:** Clarithromycin susceptibility testing result of different species.

Subspecies	Incubation days	Susceptible breakpoint	Moderately susceptible breakpoint	Resistant breakpoint	MIC range (*μ*g/mL)	MIC_50_ (*μ*g/mL)	MIC_90_ (*μ*g/mL)	Number (%) of susceptible strains	Number (%) of moderately susceptible strains	Number (%) of resistant strains
*M. abscessus *subsp.* abscessus* (*n* = 45)	14	≤2	4	≥8	1–>32	>32	>32	5 (10%)	3 (7%)	37 (83%)

*M. abscessus *subsp.* massiliense* (*n* = 25)	14	≤2	4	≥8	≤0.0625–>32	0.25	0.25	24 (96%)	0 (0%)	1 (4%)

*M. fortuitum* (*n* = 23)	3	≤2	4	≥8	≤0.0625–>32	0.5	4	21 (92%)	1 (4%)	1 (4%)

MAC (*n* = 131)	7	≤8	16	≥32	≤0.0625–>32	0.5	8	124 (95%)	1 (1%)	6 (4%)

*M. kansasii* (*n* = 34)	7	≤16	—	>16	≤0.0625–16	≤0.0625	1	34 (100%)	—	0 (0%)

**Table 4:** Clarithromycin susceptibility testing result of *M. chelonei *and *M. fuerth*.

Species	Incubation days	Isolate's number	MIC value	DST result
*M. chelonei* (*n* = 5)	3	2 3 13 14 17	≤0.0625 ≤0.0625 ≤0.0625 0.125 ≤0.0625	Susceptible Susceptible Susceptible Susceptible Susceptible

*M. fuerth* (*n* = 2)	3	12 25	0.25 ≤0.0625	Susceptible Susceptible

**Table 5:** The relationship between clarithromycin's resistance and* erm(41)*.

Subspecies	MIC value of day 14 (*μ*g/mL)	Number	DST result	The base at size 28 of *erm(41) *
*M. abscessus *subsp.* abscessus* (*n* = 40)	>32	*n* = 34	R	T
	8	*n* = 1	R	C
	1–4	*n* = 5	S	C
